# Antifungal and Anti-Virulent Activity of *Origanum majorana* L. Essential Oil on *Candida albicans* and In Vivo Toxicity in the *Galleria mellonella* Larval Model

**DOI:** 10.3390/molecules27030663

**Published:** 2022-01-20

**Authors:** Banu Kaskatepe, Sinem Aslan Erdem, Sukran Ozturk, Zehra Safi Oz, Eldan Subasi, Mehmet Koyuncu, Josipa Vlainić, Ivan Kosalec

**Affiliations:** 1 Department of Pharmaceutical Microbiology, Faculty of Pharmacy, Ankara University, Ankara 06560, Turkey; bkaskatepe@ankara.edu.tr; 2 Department of Pharmacognosy, Faculty of Pharmacy, Ankara University, Ankara 06560, Turkey; sinemaslanerdem@yahoo.com; 3 Department of Pharmaceutical Microbiology, Faculty of Pharmacy, Zonguldak Bulent Ecevit University, Zonguldak 67100, Turkey; sukranozturk79@gmail.com; 4 Department of Medical Biology, Faculty of Medicine, Zonguldak Bulent Ecevit University, Zonguldak 67100, Turkey; safizehra@yahoo.com; 5 Microbiology Laboratory of Application and Research Hospital, Zonguldak Bulent Ecevit University, Zonguldak 67100, Turkey; e_ldan@hotmail.com; 6 Department of Pharmaceutical Botany, Faculty of Pharmacy, Cyprus International University, Lefkosa 99258, Turkey; mkoyuncu@ciu.edu.tr; 7 Ruđer Bošković Institute, 10000 Zagreb, Croatia; josipa.vlainic@irb.hr; 8 Faculty of Pharmacy and Biochemistry, Institute for Microbiology, University of Zagreb, 10000 Zagreb, Croatia

**Keywords:** virulence factors, CSH, biofilm, germ-tube, marjoram, carvacrol

## Abstract

The aim of this study was to investigate and compare in detail both the antifungal activity in vitro (with planktonic and biofilm-forming cells) and the essential oil composition (EOs) of naturally growing (OMN) and cultivated (OMC) samples of *Origanum majorana* L. (marjoram). The essential oil composition was analyzed using GC-MS. The major constituent of both EOs was carvacrol: 75.3% and 84%, respectively. Both essential oils showed high antifungal activity against clinically relevant *Candida* spp. with IC_50_ and IC_90_ less than or equal to 0.5 µg mL^−1^ and inhibition of biofilm with a concentration of 3.5 µg mL^−1^ or less. Cultivated marjoram oil showed higher anti-biofilm activity against *C. albicans*. In addition, OMC showed greater inhibition of germ-tube formation (inhibition by 83% in Spider media), the major virulence factor of *C. albicans* at a concentration of 0.125 µg mL^−1^. Both EOs modulated cell surface hydrophobicity (CSH), but OMN proved to be more active with a CSH% up to 58.41%. The efficacy of *O. majorana* EOs was also investigated using *Galleria mellonella* larvae as a model. It was observed that while the larvae of the control group infected with *C. albicans* (6.0 × 10^8^ cells) and not receiving treatment died in the controls carried out after 24 h, all larvae in the infected treatment group survived at the end of the 96th hour. When the treatment group and the infected group were evaluated in terms of vital activities, it was found that the difference was statistically significant (*p* < 0.001). The infection of larvae with *C. albicans* and the effects of *O. majorana* EOs on the hemocytes of the model organism and the blastospores of *C. albicans* were evaluated by light microscopy on slides stained with Giemsa. Cytological examination in the treatment group revealed that *C. albicans* blastospores were phagocytosed and morphological changes occurred in hemocytes. Our results indicated that the essential oil of both samples showed strong antifungal activities against planktonic and biofilm-forming *C. albicans* cells and also had an influence on putative virulence factors (germ-tube formation and its length and on CSH).

## 1. Introduction

Herbs and spices are very well known over the world and have been used since ancient times for flavoring, coloring, and preserving food, as well as for medicinal and cosmetic purposes [1,2]. Aromatic plants are effective and alternative antimicrobials; most of them are classified as generally recognized as safe by the FDA [3,4]. Essential oils (EOs) are known for their diverse and crucial bioactivities, but they mainly have bactericidal, virucidal, and fungicidal properties [5,6,7]. Several studies have demonstrated the antimicrobial activity of EOs even against multidrug-resistant bacteria. Moreover, EOs have been used to disinfect hospitals [5]. The genus *Origanum*, which belongs to Lamiaceae, is known to have been used as a spice since ancient times. It is represented by 52 species all over the world, 32 taxons of which are found in Turkey. *Origanum* spp. are of great commercial importance both worldwide and in Turkey [6,8,9,10]. Interestingly, the chemical composition of the essential oil of *O. majorana* L, according to studies on *O. majorana* (marjoram) essential oil, shows that the ingredients that comprise the oil have great differences from each other. Tabanca et al. [11] studied the essential oil of *O. majorana* from four different locations and found that *cis*-sabinene hydrate (30–44%) was the main constituent of all samples collected in Turkey, while carvacrol (52.5–79.5%) was found to be the main constituent of *O. majorana* essential oil in three distinctive studies from Turkey [12,13,14]. Chaves et al. [15] found pulegone (57.05%) as the main constituent of the essential oil of *O. majorana* from Brazil. Moreover, terpinene-4-ol and linalool were found as the major compounds of Egyptian- and Moroccan-originated essential oils of marjoram, respectively [16,17]. Therefore, any finding regarding the elucidation of the essential oil composition can be accepted as original for marjoram *O. majorana* (marjoram) as an important traditional aromatic plant worldwide with a wide range of traditional uses in respiratory problems, fever, hypertension, diabetes, cough, sore throat, menstrual pain, digestive ailments, dental pain, gum diseases, flu, and so on, as well as used as spice in cuisine [18]. *O. majorana*’s volatile oil was noticed to have substantial antibacterial, antifungal, antioxidant, and cytotoxic properties [19,20]. Studies revealed that the essential oil of *O. majorana* is effective against *Staphylococcus epidermidis*, *Salmonella enteritidis*, *Salmonella typhimurium*, *Escherichia coli*, *Klebsiella pneumoniae*, *Pseudomonas aeruginosa*, *Citrobacter freundii*, *Proteus mirabilis*, and *Candida albicans* [18,20,21]. Marjoram essential oil was found to inhibit colon cancer by inducing protective autophagy and apoptotic cell death in vitro [22]. The oil has been examined in vivo for antidiabetic and antiulcer activity and has been found to be significantly effective [23]. Although the antimicrobial properties of marjoram essential oil are well known and have been studied, no research work has been found on its inhibitory effect on the virulence of *Candida albicans* and the larval model of *Galleria mellonella*. The moth *G. mellonella*, which naturally invades beehives, is a member of the subfamily Galleriinae, which belongs to the family Pyralidae in the order Lepidoptera [24]. *G. mellonella* is one of the preferred in vivo models to determine the pathogenesis of infections and the virulence factors of the microorganisms and to identify effective treatment options. It is also used in areas such as determining fungal and bacterial loads and evaluating antimicrobial peptides [25,26]. In addition, the immunity of this model consists of cellular and humoral defenses similar to those of mammals, providing an advantage in monitoring the infection process. The ability of these larvae to survive at 15–37 °C is very important [27,28]. Since the expression of virulence factors of many pathogenic microorganisms that threaten human health occurs at 37 °C, this fact brings *G. mellonella* larvae to the forefront in establishing treatment protocols against infectious diseases [24,29,30]. The hemolymph of *G. mellonella* larvae contains different types of immune system cells called hemocytes. In infection model studies, hemolymph is a valuable material to demonstrate the immune response against infections and the interaction between host and pathogen. It has been reported that hemolymph contains six different types of hemocytes, and the hemocyte density and hemocyte types differ depending on the infectious agent [31,32]. It is emphasized that this organism is a reliable model for both bacterial and fungal pathogenicity studies [33,34,35,36].

The aim of the present study was to compare and clarify the chemical composition of the essential oil of naturally growing and cultured *O. majorana* using GC-MS and to determine the antifungal activity on both planktonic and biofilm-forming yeast cells. The ability of essential oils to modulate the putative virulence factors of *C. albicans in vitro*, such as germ-tube formation and its length and cell surface hydrophobicity (CSH), was investigated. In this study, an infection model of *C. albicans* on *G. mellonella* was performed to determine the effect of *O. majorana* essential oil.

## 2. Results

### 2.1. Chemical Composition of the Essential Oils

The yield of EOs for OMN and OMC was 6.3% and 5.3% *v*/*w*, respectively. After obtaining essential oils, they were investigated by GC-MS to reveal the composition of the oils. The compositions of the oils analyzed are given in Table 1. Both oils were rich in oxygenated monoterpenes, namely 79.2% for OMN and 88.7% for OMC. Monoterpene hydrocarbons were also present in significant amounts in the oils, 19.5% and 10.8%, respectively. Sesquiterpene hydrocarbons were not found in any of the samples, as well as only a very small amount of caryophyllene oxide, an oxygenated sesquiterpene found in OMC. The main constituents of the two EOs from OMN and OMC were carvacrol, accounting for 75.3% and 84%, and *p*-cymene, accounting for 7.1% and 4.8%, respectively.

### 2.2. Antifungal Activity—Microdilution Assay Results

Both essential oils showed strong antifungal activity against all *Candida* spp. tested with the IC_50_ ranging from <0.0156 µg mL^−1^ to 0.25 µg mL^−1^ and with slightly higher IC_90_ values ranging up to 0.5 µg mL^−1^. The most sensitive species was *C. dubliniensis* MFBF 11098 with the lowest IC_50_ and IC_90_ values compared to the other *Candida* spp. No differences were observed in the IC_50_ and IC_90_ values between the essential oils of natural (OMN) and cultivated (OMC) specimens of *O. majorana*. Both fluconazole-sensitive and fluconazole-resistant clinical *C. albicans* strains were sensitive to the essential oils of *O. majorana*. The results of the antifungal activity of the essential oils and amphotericin B as the control are shown in Table 2.

### 2.3. Biofilm Inhibition Assay

**Figure 1:**
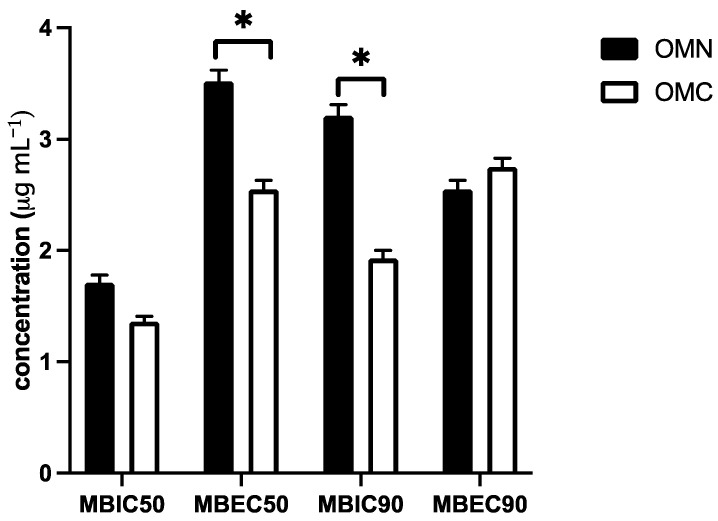
Comparison of the minimal biofilm inhibition (MBIC) and biofilm eradication concentration (MBEC) between essential oils obtained from cultivated (OMC) and naturally harvested (OMN) *O. majorana* (mean ± SD, *n* = 3, * *p* < 0.05).

In addition to the antifungal activities of marjoram EOs in the assay with planktonic *Candida albicans* ATCC 90,023 cells, the biofilm inhibition assay was also performed. Comparing the minimum biofilm inhibition (MBIC) and biofilm eradication concentration (MBEC) between the EOs, it was found that the vegetable oils showed anti-biofilm activity in vitro at a concentration higher than the IC_50_/IC_90_ where the planktonic *C. albicans* cells were present. However, OMC marjoram EO inhibited biofilm with a lower MBIC_90_ concentration of 1.93 ± 0.05 µg mL^−1^ than OMN EO with an MBIC_90_ of 3.21 ± 0.07 µg mL^−1^ (*p* < 0.05) (Figure 1). The results of MBEC_50_ between OMN and OMC EOs also showed differences in biofilm inhibition activity with lower values (*p* < 0.05) of OMC (2.55 ± 0.08 µg mL^−1^) than OMN (3.52 ± 0.1 µg mL^−1^). The differences in biofilm-inhibitory activity between OMN and OMC resulted in MBEC_50_ and MBIC_90_ comparison where OMC EO showed more potent activity with a lower inhibitory and eradication concentration than OMN. The in vitro activity against the biofilm formed by *C. albicans* cells showed that the essential oil of OMC exhibited stronger in vitro antibiofilm activity than OMN (Figure 1).

### 2.4. Germ-Tube Inhibition Assay and Modulation of Germ-Tube Length

The percentage inhibition of germination in three different media was determined in comparison to the negative control (media without essential oils). OMC and OMN EOs inhibited germ-tube formation in different ratios. The results of germ-tube inhibition of *C. albicans* after treatment with two different concentrations of OMN and OMC are shown in Table 3. The best results in the inhibition of germ-tube formation were obtained with Spider media (from 75% to 83%, respectively). The results suggested that marjoram EOs affect the metabolic pathway regulating the bud-to-hyphae transition *in vitro* of *C. albicans*; thus, the EO is involved in the inhibition of one of the major virulence factors of *C. albicans* (Table 3).

**Figure 2:**
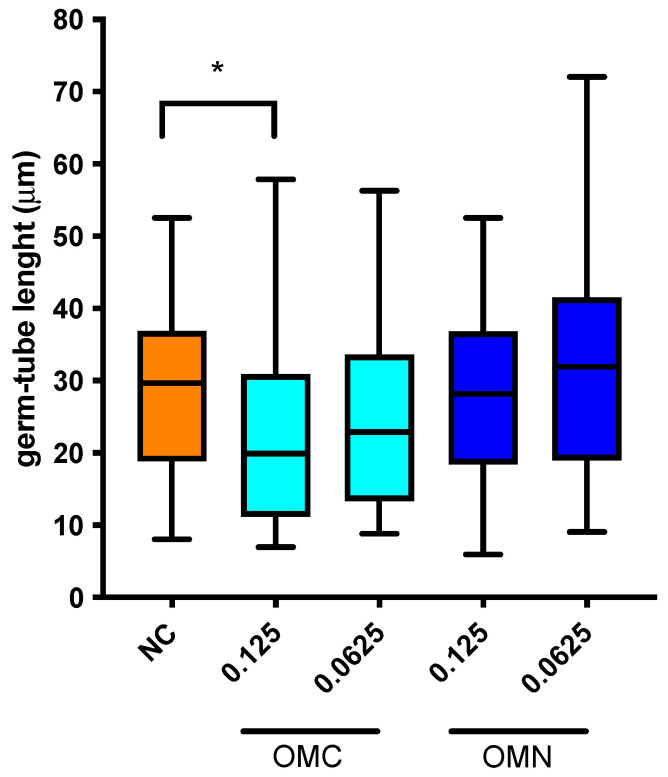
The inhibition of the germ-tube length of *C. albicans* ATCC 90023 treated with cultivated (OMC) and naturally harvested (OMN) *O. majorana* essential oils at two concentrations (the results were obtained on 100 cells per every group, shown as the mean ± SD with the minimal and maximal length in µm; * means significant differences (*p* < 0.05) in comparison to untreated cells (NC, negative control).

Based on the results of the germ-tube inhibition test obtained in three different hypha-induced media with two different concentrations of *O. majorana* OMN and OMC EOs (Table 3), we investigated the effect of both EOs on the length of the germ-tube. The results presented in Figure 2 show that only the OMC EOs at a concentration of 0.125 µg mL^−1^ significantly reduced the germ-tube length of *C. albicans* ATCC 90023 in the medium RPMI 1640 with an addition of 10% (*v*/*v*) FBS. Thus, OMC was able to modulate both the formation of germ-tubes and their length, contributing to the inhibition of one of the most important virulence factors of *C. albicans*.

### 2.5. Modulation of Cell Surface Hydrophobicity

The results showed that both EOs at concentrations below the MIC (0.125 µg mL^−1^ and 0.0625 µg mL^−1^) modulated the CSH levels of *C. albicans* after incubation at 25 °C. Compared to intact yeast cells, the essential oil of OMN showed a stronger effect on hydrophobicity with an inhibition of more than 50% (Table 4). The maximum modulation of CSH by OMC was only 25.7%, indicating a low ability of OMC to modulate cell surface hydrophobicity.

### 2.6. Defensive Response of Infected G. mellonella

**Figure 3:**
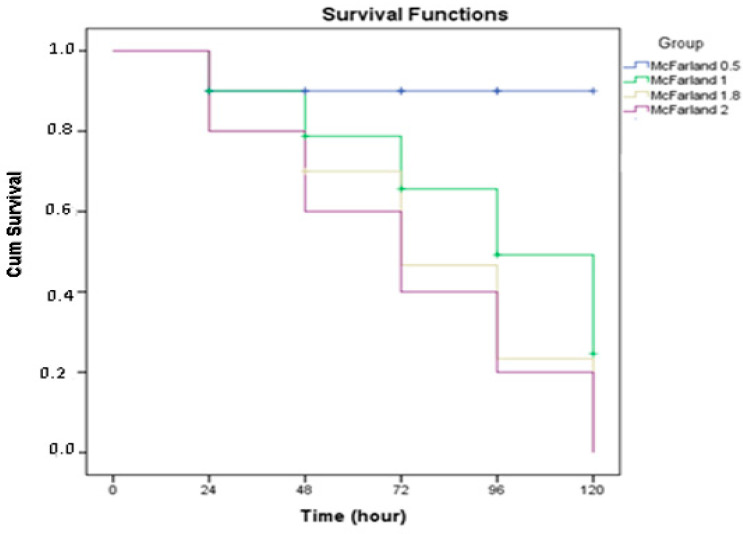
Defensive response of infected *G. mellonella*: Kaplan–Maier survival curves (±SE., *n* = 20) of larvae injected with 1.5 × 10^8^, 3.0 × 10^8^, 5.4 × 10^8^, and 6.0 × 10^8^ *C. albicans* cells. A statistically significant difference (*p* < 0.034) was found between the groups in terms of lifespan.

The sensitivity of larvae to intrahemocelic injection was dependent on the dose of the C. albicans cells. At a cell dosage of 1.5 × 10^8^ (McFarland 0.5), only 5% of the larvae died 72 h after injection (LD_5_, non-lethal dosage). At a cell dosage of 3 × 10^8^ cells (McFarland 1.0), 50% of the larvae died within 72 h after injection, and 90% of the larvae died after 72 h with the McFarland 1.8 units of yeast cells (LD_90_; lethal dose). Finally, 24 h after the injection at a cell dosage of 6.0 × 10^8^ (McFarland 2.0), all larvae died (LD_100_) (Figure 3).

### 2.7. The Effects of O. majorana EO Treatment on Infected G. mellonella

**Figure 4:**
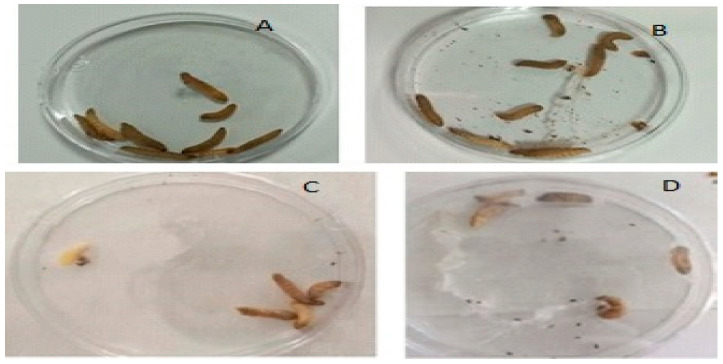
The groups of *G. mellonella* larvae: (**A**) Therapy group with 3.2 mg mL^−1^ *O. majorana* in *G. mellonella* larvae infected with an inoculum of 6.0 × 10^8^ cells of *C. albicans* ATCC 90028 treatment for 24 h, (**B**) Therapy Group 1 with 3.2 mg mL^−1^ *O. majorana* EO in *G. mellonella* larvae infected with an inoculum of 6.0 × 10^8^ cells of *C. albicans* ATCC 90028 treatment for 48 h, (**C**) Therapy Control Group 1 (3.2 mg mL^−1^ *O. majorana* EO), and (**D**) Therapy Control Group 2 (3.2 mg mL^−1^ *O. majorana* EO).

**Figure 5:**
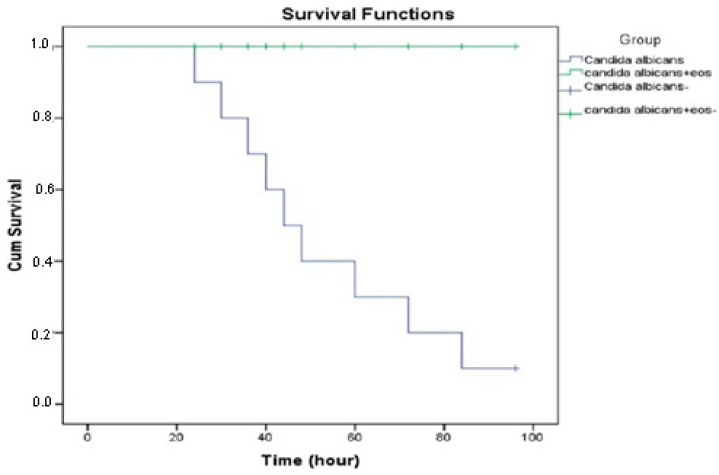
Kaplan–Meier analysis was performed to estimate survival time, and subgroups were compared using the log rank test. A *p*-value of less than 0.05 was considered statistically significant for all tests. There was a significant difference between the groups (infected groups 6.0 × 10^8^ *C. albicans* cells and treatment groups with *O. majorana* EOs) in terms of survival rates (*p* < 0.001).

Larvae infected with *C. albicans* (6.0 × 10^8^ cells) and treated with EO were examined after 24 h, 48 h, 72 h, and 96 h. The evaluation of the survival rate of larvae in the infected treatment group from the 24th hour showed that all larvae survived at the end of the 96th hour (Figure 4). The survival curves of the larvae are shown in Figure 5. The larvae of the control group infected with *C. albicans* (6.0 × 10^8^ cells) and not receiving treatment also died in the controls carried out after 24 h. When the treatment group and the infected group were evaluated in terms of vital activities, it was found that the difference was statistically significant (*p* < 0.001).

### 2.8. Cytology of G. mellonella Larvae

**Figure 6:**
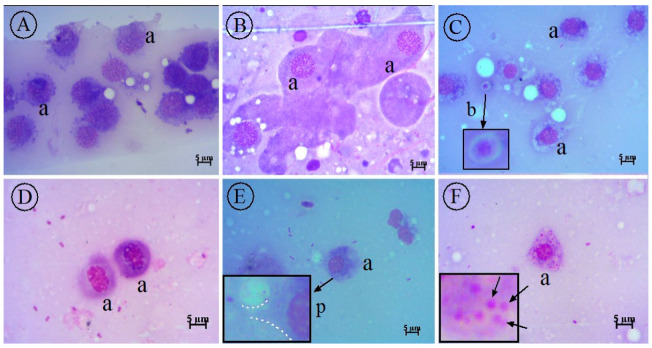
Healthy *G. mellonella* hemocytes (**A**,**B**) and their response to *C. albicans* infection (**C**) and *O. majorana* (EO) treatment (**D**,**F**) under light microscopy (Giemsa stain × 100). (**A**) Healthy *G. mellonella* hemocytes (a); (**B**) healthy *G. mellonella hemocytes* (a) treated with *O. majorana* EO; (**C**) *C. albicans* blastospore (arrow, b) and *G. mellonella* hemocytes (a); (**D**) mitotic division in *G. mellonella* hemocyte; (**E**) *G. mellonella* (a) pseudopods begin to form at the initiation of phagocytosis; (**F**) phagocyted blastospores (arrow) engulfed by *G. mellonella* hemocytes (a).

**Figure 7:**
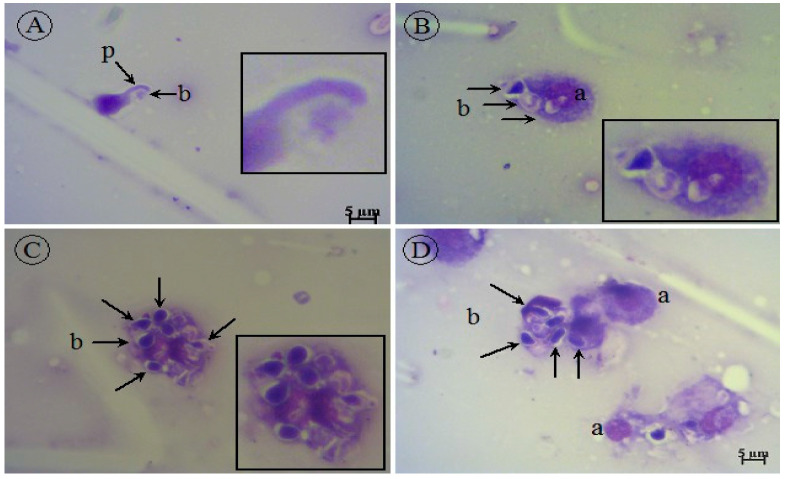
*G. mellonella* hemocytes infected with *C. albicans* and subsequently treated with *O. majorana* EO under light microscope (**A**–**D**) (MGG stain ×100). (**A**) Initial stages of phagocytosis of *C. albicans* blastospore (arrow, b) with pseudopodia (arrow, p) of *G. mellonella* (a); (**B**) continuing phagocytosis of *C. albicans* blastospores (arrow, b) by *G. mellonella* (a); (**C**) *C. albicans* blastospores (arrow, b) phagocyted by *G. mellonella*; (**D**) *C. albicans* blastospores (arrow, b) degraded after phagocytosis by *G. mellonella* (a).

In the smears, different types of hemocytes were observed in the hemolymph. Blastospores of *C. albicans* were found in the infected smears, but *C. albicans* hyphae were not found in any of the smears. Larvae with or without *C. albicans* infection treated with *O. majorana* EO are shown in Figure 6 and Figure 7. In our study, compared to the control groups, as a result of treatment with EOs, it was observed that the infected group that did not receive treatment died within the first 24 h, while the treatment group survived until the 96th hour. Cytological examination in the treatment group revealed that *C. albicans* blastospores were phagocytosed and morphological changes occurred in hemocytes (Figure 7A–D). *O. majorana* EO treatment induced the formation of pseudopods in the hemocytes of *G. mellonella* and thus increased the antifungal phagocytosis activity of the hemocytes. Since actin cytoskeleton flicker is known to be effective in pseudopod formation, it is hypothesized that *O. majorana* EO increases actin cytoskeleton polymerization in *G. mellonella* hemocytes.

## 3. Discussion

The essential oil yield of *O. majorana* species found in the literature varies in a wide range: Amor et al. [40] reported the essential oil yield as 0.97%, while Aytaç [41] found a value of 4.2%. In another study, the yield was reported as 1.2% [42]. In our study, the yields of essential oils were found to be higher, as 5.3% and 6.3%.

Both oils were rich in oxygenated monoterpenes, and the main constituents of the two EOs from OMN and OMC were carvacrol, accounting for 75.3% and 84%, and *p*-cymene, accounting for 7.1% and 4.8%, respectively. Mossa and Nawwar [43] analyzed Egyptian samples of EO and reported terpinene-4-ol, γ-terpinene, and *trans*-sabinene hydrate as major constituents. Similarly, the essential oil of *O. majorana* from Tunis was studied at different stages of growth, and again, terpinene-4-ol was found to be the major component followed by *cis*-sabinene hydrate, *trans*-sabinene hydrate, and geranyl acetate. The two studies summarized above contained very small amounts of carvacrol. In two studies, the essential oils of *O. majorana* collected from different places were investigated, and carvacrol was found to be the major constituent in amounts of 78.27–79.5% [12,40]. In another study also conducted by Tabanca et al. [11], essential oils of *O. majorana* from four different localities were investigated, and *cis*-sabinene hydrate and terpinen-4-ol were found as major constituents. Baser et al. [44] described the presence of two chemotypes of essential oils of *O. majorana*: *cis*-sabinene hydrate/terpinen-4-ol type and carvacrol/thymol chemotype. According to the results, both analyzed oils belong to the latter type.

Depending on the geographical origin, climatic conditions, part of the plant, age, and season in which the material is collected, the quality, quantity, and chemical content of essential oils may vary considerably [1]. Chemical composition, storage temperature, and pH are the factors affecting the antimicrobial activity of the oils [45]. In view of the above results on the yield and composition of the essential oils, studies on the essential oil composition and antimicrobial activity of naturally growing and cultured samples of *O. majorana* may add to the knowledge of the study.

Carvacrol was predominantly determined as the major constituent of both oils. It can be concluded that the antifungal activity is due to the high carvacrol content, as there are a number of studies demonstrating the antifungal activity of carvacrol [46,47,48,49]. When comparing wild and cultured samples, the sample from nature showed slightly higher activity; the amount of carvacrol was higher in the cultured sample, but the amounts of *p*-cymene and γ-terpinene were higher in the essential oil from nature. It has been shown that there is a synergism between thymol and *p*-cymene for antifungal activity, and it could be assumed that this synergism also applies to carvacrol and *p*-cymene. Thus, although the amount of carvacrol in the essential oil of the natural sample was lower, a higher activity was ensured due to the synergism between carvacrol and *p*-cymene with respect to the higher amount of *p*-cymene in the wild sample [46].

Our results of inhibitory activity against medicinally important *Candida* spp. showed that both samples, OMC and OMN, of essential oils had lower IC_50_/IC_90_ values compared to Tunisian samples of *O. majorana* essential oil [50]. The results of MIC values [50] ranged from 0.058 mg mL^−1^ to 0.468 mg mL^−1^ using the serial twofold microdilution broth method. However, in the above-mentioned study by Hajlaoui et al. [50], the chemical composition showed the presence of terpinen-4-ol (23.2%) and *cis*-sabinene hydrate (17.5%) as major constituents, in contrast to the carvacrol chemotype of the essential oil samples in the present study (Table 1). An older study by Sarer et al. [51] on three samples of *O. majorana* grown naturally in Turkey using GC and GC-MS showed that carvacrol was the most abundant compound with concentrations ranging from 48.4% to 73.5%, depending on the sample. Screening of antifungal activity of the samples of *O. majorana* essential oils by the disk diffusion method against *C. albicans* confirmed the antifungal activity with inhibition zones of 32 mm and 40 mm, respectively. It is interesting to highlight that Sarer et al. [51] compared the antifungal activity of samples of essential oils of *O. majorana* with carvacrol, which showed very strong activity with zones of inhibition of 40 mm against *C. albicans*. In another study by Ragab et al. [16], the antifungal activity of Egyptian essential oils of *O. majorana* obtained by microwave-assisted extraction, steam distillation, and conventional hydrodistillation were compared using the disk diffusion test. Interestingly, only the essential oil obtained by steam distillation was found to be active against *C. albicans* ATCC 10231 with inhibition zones of 30 ± 0.20 mm. The sample of Egyptian essential oil in this study was rich in terpinen-4-ol (26.72%) [16]. In the study of Charai et al. [17] on the essential oil of *O. majorana* from Morocco, linalool (32.68%) and terpinen-4-ol (22.30%) dominated the sample, and antifungal activity was tested against the yeasts *Saccharomyces cerevisiae*, *Candida utilis*, *C. lipolytica*, and *C. tropicalis*. All yeast species and strains tested were completely inhibited with 5 ppm of the essential oil using the Charai et al. [17] microdilution broth assay.

Our results showed for the first time that in addition to the antifungal activity of *O. majorana* EOs against clinically important *Candida* spp. in planktonic form, both oils also inhibited biofilm formation (Figure 1). To test the anti-biofilm activity, both EOs were added at the same time as *C. albicans* cells, so that the inhibition of yeast cell adhesion was the result of the biofilm inhibition assay. Compared to the IC_50_/IC_90_ values and MBIC_50_/MBEC_50_ and MBIC_90_/MBEC_90_ (Figure 1), both EOs showed a significantly higher concentration to inhibit cells in filamentous forms in biofilm than in planktonic forms. There were differences in anti-biofilm activity between OMN and OMC EOs. As shown by the MBIC_90_ value, OMC EO proved to be more effective in inhibiting adhesion than the OMN sample, while the same was found when the MBEC_50_ values were compared. Thus, OMN at a concentration of MBIC_90_ of 1.93 ± 0.02 μg mL^−1^ and MBEC_50_ of 2.75 ± 0.01 μg mL^−1^, statistically lower (*p* < 0.05) values of biofilm inhibition were shown than the OMN counterpart (Figure 1). These data could lead to a next stage of anti-biofilm research, such as the inhibition of mature biofilm formation [52].

Based on the very low IC levels against clinically important *Candida* spp. that were 0.5 μg mL^−1^ or less and MBIC and MBEC levels that were 6.75 μg mL^−1^ or less, we performed several *in vitro* experiments with a focus on the virulence of *O. majorana* EOs. It is well documented that *C. albicans* secretes virulence factors that promote the colonization and spread of filamentous yeast cells in tissues [53,54]. Therefore, targeting such virulence factors is an interesting strategy to control fungal infections by essential oils or their compounds [55]. There are several virulence factors of *C. albicans*, including the production of extracellular hydrolytic enzymes such as proteases, phospholipases, and hemolysins, cellular morphogenesis (bud-to-hypha transition, germ-tube formation), and the aforementioned adhesion to abiotic surfaces and to host epithelial cells, as well as cell surface hydrophobicity [56,57,58]. Therefore, we tested the modulating effect of *O. majorana* EOs on the formation of germ-tubes and the influence of EOs on the length of the formed germ-tubes. In addition, the inhibitory effect of EOs on the adhesion of *C. albicans* cells was tested by modulating the hydrophobicity of the cell surface. As can be seen in Table 3 and Figure 3, both EOs modulated the formation of germ-tubes and the length of hyphal buds formed. The inhibition of the bud-to-hyphal transition of *C. albicans* was studied in four different media capable of inducing budding. The composition of the media correlates with different hyphal-inducing signals and different pathways in *C. albicans* cells [53]. Although both EOs showed a modulatory effect on germ-tube formation, it was clear that both oils at lower concentrations strongly inhibited germ-tube formation in nutrient-poor Spider media containing 1% mannitol as the substrate (Table 3). At the lowest concentration tested (0.0625 µg mL^−1^), OMN and OMC EOs inhibited the filamentation of *C. albicans* cells in Spider hyphal-inducing media by 81 ± 2.70% and 83 ± 3.75%, respectively, which was the strongest inhibition (*p* < 0.05) compared to the other hyphal-inducing media. The composition of the Spider media induces the transition of *C. albicans* unicellular blastospores to budding cells, and this transition is mediated by the cAMP-dependent protein kinase (cAMP-PKA) pathway [59]. In view of this, both EOs contain a compound that strongly inhibits budding transition via the cAMP-PKS pathway, but other pathways are also affected, albeit to a lesser extent (Table 3).

The length of germ-tubes was also significantly altered by *O. majorana* EOs, and the reduction in germ-tube length may be directly related to the inhibition of *C. albicans* virulence. Compared with untreated *C. albicans* cells (negative control), the length of germ-tubes was significantly decreased only in the group of OMC EOs, while the other samples of *O. majorana* EO had no effect on the length of germ-tubes (Figure 2). This activity may also be related to the data from the germ-tube assay, which suggests that the OMC sample of *O. majorana* EO directly modulates one of the most important virulence factors of *C. albicans*, hyphal transition. Some EOs containing carvacrol, such as oregano (*Origanum vulgare* L.) essential oil, inhibited the expansion of mycelia and the formation of germ-tubes of *C. albicans* cells, one of the most important virulence factors of this medically important yeast [60]. Since the essential oils investigated in this study are of the carvacrol-chemotype, it could be hypothesized that the most abundant compounds in the essential oil are responsible for inhibiting the virulence factors of *C. albicans*.

*C. albicans* cells showed specific adhesion receptor interactions with their own cell surface and surfaces of the environment, which can be abiotic (catheters, implants, etc.) or biotic, such as epithelial cells and tissues. It has been demonstrated that hydrophobic cells of *C. albicans* promote adherence, so the modulation of cell surface hydrophobicity (CSH) could be one of the strategies to influence the virulence of yeast. The modulation of CSH also has an impact on biofilm formation, as hydrophobic strains have stronger adhesion to surfaces and consequently a better ability to form biofilms [61]. The EOs of *O. majorana* stimulated changes in CSH levels (Table 4). Our results showed that the EO of OMN is the best inducer of CSH levels by inhibiting hydrophobicity from 52.6% to 58.41%, in comparison with untreated cells (Table 4).

*C. albicans* cells when injected into the larval hemocele can kill *G. mellonella* [62]. It is well known that hemocytes have an effective role in phagocytosis-mediated protection against pathogens [31,32]. It has been reported that *C. albicans*, hidden in the biofilm structure, play a role in inducing the release of different cytokines by the host and damaging the immune response against phagocytic cells [63].

Vertyporokh et al. [62] observed survival in larvae with a 2 × 10^4^ and a 2 × 10^5^ density of *C. albicans* cell suspension injected into the hemocele and reached the lethal dose with 2 × 10^6^ at the end of the 24th hour. In our study, the lethal dose was reached at the end of the 24th hour by using a *C. albicans* suspension with a density of 6 × 10^8^ cells.

In our literature survey, no study was found related to the effect of *O. majorana* EOs on the *G. mellonella* larval model. *O. majorana* EO induced pseudopodia formation in *G. mellonella* hemocytes (Figure 7A,E). The observation of hemocytes containing phagocyted particles in Figure 7F suggested that *O. majorana* increased the phagocytic activity of hemocytes (Figure 5F and Figure 6). The virulence of *C. albicans* includes filamentation, proteinases, adherence proteins, and biofilm formation [64]. The absence of *C. albicans* hyphae in infected and treated smears may indicate that *G. mellonella* affects the filamentation of *C. albicans* in the defense between *C. albicans* and *G. mellonella*.

The *G. mellonella* moth that naturally invades beehives is a member of the *Galleriinae* subfamily belonging to the *Pyralidae* family of the Lepidopteran order [24]. In infection model studies, hemolymph is a valuable material in demonstrating the immune response against infection and host–pathogen interaction. It has been reported that hemolymph contains six different types of hemocytes, and the hemocyte density and hemocyte types differ depending on the infectious agents [31,32]. It is emphasized that this organism is a reliable model in both bacterial and fungal pathogenicity studies [33,34,35,36]. In this study, the infection model of *C. albicans* was formed on *G. mellonella* to determine the effect of OMN.

## 4. Materials and Methods

### 4.1. Plant Material

The cultivated sample of *O. majorana* L. was collected from a farm in Anamur, Mersin, Turkey. The naturally growing specimens of *O. majorana* were collected near Anamur, Mersin, in 2012. The voucher specimens of the naturally growing and cultivated samples were identified by Professor Mehmet Koyuncu, Ph.D., and deposited in the Herbarium of the Faculty of Pharmacy, Ankara University (AEF 26261 and AEF 26262, respectively).

### 4.2. Distillation of Essential Oils

The essential oils were obtained from air-dried and powdered plant materials (100 g) subjected to hydro-distillation using the Clevenger apparatus for three hours.

### 4.3. GC and GC-MS Analysis of Essential Oils

GC analysis of the *O. majorana* EOs was performed with Agilent GC analysis using the Agilent 6890N Network GC and 5973 Network mass selective detector GC-MS system previously used by our research group [12,13,37,38,39]. The analysis was performed using an HP-Innowax column (60.0 m × 0.25 mm × 0.25 mm) (Agilent Technologies, CA, USA) and helium as the carrier gas (1.2 mL min^−1^). The operating conditions were as follows: the oven temperature was set at 60 °C for 10 min after injection, then increased to 220 °C with a heating ramp of 4 °C/min for 10 min, and then increased to 240 °C with a heating ramp of 1 °C/min without holding; the injector and detector (FID) temperatures were 250 °C; the split ratio was set at 20:1; the injection volume was 2.0 μL. The MS conditions were as follows: ionization energy 70 eV; ion source temperature 280 °C; interface temperature 250 °C; mass range 34–450 atomic mass units. The compounds were identified by comparing their relative retention indices and mass spectra with the corresponding literature [56] and by comparing their mass spectra with the Wiley and NIST libraries. The percentages of the components were calculated from the GC peak areas using the normalization method. The GC analyses were duplicated.

### 4.4. Antimicrobial Susceptibility Testing

All strains, including *C. albicans* ATCC 90028, *C. albicans* (fluconazole sensitive) MFBF 10778, *C. albicans* (fluconazole resistant) MFBF 11100, *C. tropicalis* ATCC 750, *C. krusei* ATCC 14243, *C. dubliniensis* MFBF 11098, were acquired from the stock cultures at the Institute of Microbiology, Faculty of Pharmacy and Biochemistry, University of Zagreb, and cultured on Sabouraud 2% (*w*/*v*) glucose agar (SDA) (Merck, Darmstadt, Germany) prior to analysis. The minimum inhibitory concentrations of EOs were determined by serial microdilution in RPMI 1640 broth containing 2% (*w*/*v*) glucose in sterile flat-bottomed 96-well microtiter plates ranging from 4 µg mL^−1^ to 0.0156 µg mL^−1^, according to Clinical and Laboratory Standards Institute guidelines [65]. Plates were incubated aerobically in the dark (24 h, 35 °C). The MIC was determined as the minimum concentration of essential oil allowing no more than 10% growth (IC_90_) and more than 50% (IC_50_) of microorganisms after re-incubation of a 10 µL sample with a loop from each dilution inoculated on Sabouraud 2% (*w*/*v*) glucose agar for 48 h at 35 °C [37]. Amphotericin B (Sigma-Aldrich, Darmstadt, Germany) was used as a control.

### 4.5. Inhibition of Biofilm Formation

The essential oils (OMN and OMC) were tested for their anti-biofilm activity according to Zorić et al. [66]. Briefly, the effect of *O. majorana* EOs on biofilm formation was evaluated using inoculum suspensions of fresh cultures of the *C. albicans* ATCC 90028 strain adjusted to 0.5 McFarland units (nephelometer, IKA, Staufen, Germany). A further 1:10 dilution in RPMI 1640 (Sigma-Aldrich, Germany) containing 2% (*w*/*v*) glucose was made before seeding into sterile 96-well flat-bottomed tissue plates (TPP, Trasadingen, Switzerland) pre-treated for 2 h with fetal bovine serum (Sigma-Aldrich, Germany). The investigated compounds were tested in a concentration range of 10–0.78125 μg mL^−1^. The EOs were added at the same time that the *C. albicans* cells were plated out. After another 48 h of incubation at 37 °C, under aerobic conditions and in the dark, the wells were aspirated and washed with phosphate-buffered solution (PBS, Sigma-Aldrich, Germany). The adherent microbial cells were fixed with methanol and stained with crystal violet (0.5%, *w/v*, in methanol). The results were obtained by measuring the absorbance at 540 nm using a microplate reader (Labsystems iEMS Reader, Helsinki, Finland). The minimum biofilm inhibition concentration (MBIC_50_) and minimum biofilm eradication concentration (MBEC_50_) values represent the lowest dilutions of the compounds at which microbial growth was inhibited by 50% or eliminated by 50% compared to the untreated control. All tests were performed in triplicate, and the means and standard deviations (SD) were calculated.

### 4.6. Inhibition of Germ-Tube Formation and the Length of Germinated Cells

The strain *C. albicans* ATCC 90028 was used for the germination inhibition test, which was performed with a slight modification of the method of Zuzarte et al. [67]. The hyphal-inducing media used in the present study were: (i) yeast potato glucose (YPG) media containing 10% (*v*/*v*) fetal bovine serum, (ii) Spider (1% tryptic soy broth, 1% (*w*/*v*) mannitol, 2% (*w*/*v*) K_2_HPO_4_), (iii) N-acetyl-D-glucosamine media (0.5% (*w*/*v*) N-acetyl-D-glucosamine, 0.5% (*w*/*v*) peptone, 0.3% (*w*/*v*) KH_2_PO_4_, 0.05% (*v*/*v*)). Briefly, 30 µL of inoculum suspension in physiological saline containing 2 McFarland units (approximately 6 × 10^8^ CFU mL^−1^) was added to 170 µL of hyphal-inducing media containing 0.0625 µg mL^−1^ or 0.125 µg mL^−1^ *O. majorana* EOs (OMN and OMC). Untreated yeast cells served as the negative control. Samples were incubated at 37 °C for 3 h in an orbital shaker (ES-20, Grant-bio, Cambridgeshire, UK) at 100 rpm under air cooling. Using a Dinocapture^®^ camera and software program 2.0 Version 1.5.6. Fifty cells were counted in each sample and expressed as the percentage of inhibition of germ-tube formation using phase contrast microscopy.

Using the same software, the length of germ-tubes formed of *C. albicans* ATCC 90028 in RPMI 1640 with the addition of 2% *w/v* glucose was measured after 4 h of aerobic incubation at 37 °C in an orbital shaker (ES-20, Grant-bio, Cambridgeshire, UK) at 100 rpm. One-hundred cells were measured, and the length of the formatted germ-tube is expressed as the mean with the standard deviation (SD), minimum and maximum values. Untreated *C. albicans* ATCC 90028 cells were used as a negative control. One-way comparison test ANOVA and Dunnett’s multiple comparison test were used to compare the treated groups with the negative control (untreated) cells.

### 4.7. Modulation of Cell Surface Hydrophobicity 

The activity of EOs (OMC, OMN) on the CSH levels of *C. albicans* ATCC 90028 was evaluated using the method of Ishida et al. [68]. For this purpose, the inoculum suspension (10^8^ CFU mL^−1^) was prepared with a fresh culture of *C. albicans* cells in Sabouraud 2% (*w*/*v*) glucose broth (Merck, Germany) and washed twice with PBS. Yeast cells were exposed to two concentrations of EOs and incubated in PBS for 24 h at 25 °C under the exclusion of air in the dark. Untreated yeast cells served as controls. After incubation, xylene (Sigma-Aldrich, Darmstadt, Germany) was added to the PBS at a ratio of 1:1 (*v*/*v*) and vortexed vigorously for 30 s, and after separation of the two phases at room temperature for 10 min, the aqueous phase of the test samples and control was measured in a 96-well microplate reader (LabSystems IEMS Reader, Helsinki, Finland) at 620 nm. The hydrophobicity index (CSH index) was quantified using the equation:

HI = [(A_control_ − A_test_)/A_control_] × 100

where A_control_ is the optical density before the experiment and A_test_ is the optical density after the treatment.

The inhibition of CSH was calculated as the modulation of the hydrophobicity index of the treated samples (HI_test_) compared to the untreated yeast cells (HI_control_) and expressed as a percentage using the equation:

Inhibition% = 100 − [(100 × HI_test_)/HI_control_]

### 4.8. In Vivo Effect of O. majorana Essential Oil on G. mellonella Larvae

#### 4.8.1. Determination of Minimum Lethal Concentration

The in vivo toxicity test was performed on larvae as previously described by Wijesinghe [69]. The test and the control groups of the larvae (*n* = 10) were selected and kept in separate petri dishes. Larvae with dark pigmentations or color changes including significant melanization on their bodies and larvae other than 0.2–0.3 g body weight were not included in the study. EO dilutions (concentrations ranging from 0.2–10 mg mL^−1^) of *O. majorana* were prepared in a combination of sterile PBS and polysorbate 80 (Tween^®^ 80). The left foreleg area of the larvae to be injected was first sterilized with 70% ethanol, and a 10 µL EO dilution was injected into the hemocele from the last left anterior leg of the larvae using a 1 mL insulin injector. Larvae were taken to petri dishes and kept at 28 °C. Then, 0.05% *v*/*v* polysorbate 80 (10 μL) and 10 μL of sterile PBS were applied to the negative control group (*n* = 10). The vitality of the larvae, body color, ability to form a cocoon, and the presence or absence of body movement were monitored and evaluated at the 24th, 48th, 72th, and 96th hours. The experiment was repeated twice.

#### 4.8.2. Survival Assay of *G. mellonella* Larvae Infected with *C. albicans*

*G. mellonella* was grown on a natural diet with honeybee nest remains at 28 °C in a dark environment. Late-instar larvae weighing about 0.2–0.3 g were used for the test. *C. albicans* ATCC 90028 strain was incubated in Sabouraud 2% (*w*/*v*) glucose agar (SDA) at 37 °C. The lethal dose determination trials were completed by injecting 10 µL of the fungal suspension prepared according to McFarland 0.5, 1.0, 1.8, and 2.0 with an insulin injector. The larvae were left to incubate at 37 °C in sterile petri dishes. As a result of the evaluations after 24 h, 48 h, and 72 h, it was decided that McFarland 2.0. turbidity was appropriate for the infection model of larvae with *C. albicans* [39,64].

#### 4.8.3. Determination of Survival Curves and Health Index Score

The number of viable larvae was counted from 24 h to 72 h after the injection of 10 µL *C. albicans* (6 × 10^8^ CFU mL^−1^) to the larvae. Larvae were considered dead when they showed no signs of movement when touched. Results are given using the Kaplan–Meier estimator [62]. The scoring system based on the physical condition of the larva, suggested by Loh et al. [35], was used for the health index. In this method, where a healthy larva obtains a maximum of ten points, the index includes the sum of the scores given to four criteria including survival, mobility, degree of melanization, and ability to produce cocoons [70].

#### 4.8.4. The Effects of *O. majorana* EO on the *G. mellonella* Infected with *C. albicans*

The larvae (*n* = 10), which were infected by injecting a 10 µL *C. albicans* (ATCC 90028) suspension, prepared equivalent to McFarland 2.0, with a sterile insulin injector from the left foreleg area, were incubated at 37 °C for 2 h. After the incubation period, an injection of 10 µL was performed from the right foreleg with the EO treatment dose determined as 3.2 mg mL^−1^ and incubated at 37 °C. The activity, cocoon formation, melanization, and survival scores of the larvae were evaluated at the 24th, 48th, 72th, and 96th hours.

#### 4.8.5. Hemolymph Collection and Preparation of *G. mellonella* Slides

For cytologic examination, four types of slides were prepared (larvae, larvae + *O. majorana* EO, larvae + *C. albicans*, larvae + *C. albicans* + *O. majorana* EO) and stained with Giemsa and May–Grünwald–Giemsa (MGG). For microscopic preparations, samples were kept at room temperature. For 10 min, each larva was placed on ice and then sterilized using 70% ethanol containing a piece of cotton before hemolymph collection, after which the abdomen cuticle of the larvae was pierced with a sterile microneedle and bled directly on to a glass slide and allowed to dry [69].

For Giemsa staining, air-dried slides were fixed in methanol for 10 min and stained 10 min with Giemsa. After the staining, the slides were rapidly washed with phosphate-buffered saline [71]. For May–Grünwald–Giemsa (MGG) staining, the slides were allowed to dry for 30 min and stained with May–Grünwald–Giemsa for 2.5 h. After May Grunwald staining, smears were rinsed with distilled water and stained with Giemsa solution (1:4 dilution of Giemsa stain with distilled water) for 10 min. The slides were rinsed and dried. The slides were places in xylene for 1 min and covered with a coverslip.

Each slide was examined under a light microscope, Olympus BX 51 (Olympus, Tokyo, Japan), at 40× and 100× magnification. The images were acquired with a photo camera (Axio Lab.A1 Carl Zeiss and Axio Cam ERc5s).

### 4.9. Statistical Analysis

Statistical analyses were performed with the SPSS 19.0 software program. Groups were compared with the Kruskal–Wallis test for the survival function. Dunnett’s test was used as the post-hoc test after the Kruskal–Wallis test. Kaplan–Meier analysis was tested for estimating the survival time, and subgroups were compared with the log rank test. For all tests, *p* < 0.05 was considered as statistically significant.

## 5. Conclusions

In terms of chemical composition, the results were quite similar when compared to the EOs of naturally growing and cultivated *O. majorana* species. Therefore, it can be concluded that this species can be grown where it is cultivated and marketed safely. Our results in terms of antifungal activity and efficacy on virulence modulation activity suggested that the essential oil of *O. majorana* has potential for the development of alternative biological agents for the pharmaceutical industry. In addition, this study investigated the efficacy of *O. majorana* essential oils on *C. albicans* infection using an in vivo larval model. It has been shown that the larval model can be used as a reliable, inexpensive, and easy-to-use model compared to other in vivo models in studying the effects of fungal infection on the host cell. It has been observed that the use of *O. majorana* EO enhances immune defense by increasing phagocytic activity in larvae infected with *C. albicans*. Based on these results, it can be assumed that *O. majorana* EO can be used for supportive treatment of individuals at risk of *C. albicans* infection or for whom treatment has not achieved sufficient success.

## Figures and Tables

**Table 1:** Composition of the natural and cultured *O. majorana* EOs.

	RRI ^a^	Compound	% Amount	
			OMN	OMC
1	1015	Methyl 2-methylbutyrate	0.1	0.2
2	1023	Methyl isovalerate	-	tr ^b^
3	1033	*α*-Pinene	0.5	0.3
4	1034	*α*-Thujene	1.2	0.8
5	1070	Camphene	0.2	0.1
6	1107	*β*-Pinene	0.1	0.1
7	1122	Sabinene	0.1	-
8	1148	*δ*-3-Carene	0.1	0.1
9	1158	*β*-Myrcene	1.9	1.1
10	1162	*α*-Phellandrene	0.3	0.3
11	1162	*α*-Terpinene	1.6	0.7
12	1197	Limonene	0.3	0.2
13	1204	1,8-Cineole	0.2	0.1
14	1207	*β*-Phellandrene	0.5	0.2
15	1226	(Z)-*β*-Ocimene	0.2	-
16	1242	*γ*-Terpinene	5.1	2.0
17	1262	*p*-Cymene	7.1	4.8
18	1274	*α*-Terpinolene	0.3	0.1
19	1402	1-Octene-3-ol	0.3	0.1
20	1425	*trans*-Sabinene hydrate	0.2	0.5
21	1482	Camphor	0.5	-
22	1499	Linalool	0.3	0.4
23	1508	*cis*-Sabinene hydrate	0.1	0.2
24	1577	Terpinen-4-ol	1.1	1.0
25	1596	*trans*-Dihydrocarvone	0.1	0.2
26	1615	*cis*- Dihydrocarvone	-	0.1
27	1673	α-Terpineol	0.1	0.7
28	1682	Borneol	0.5	0.6
29	1715	Carvone	0.2	0.2
30	1820	*p*-Cymen-8-ol	-	0.1
31	2000	Caryophyllene oxide	-	0.1
32	2147	Thymol	0.6	0.6
33	2186	Carvacrol	75.3	84.0
		**Monoterpene hydrocarbons**	**19.5**	**10.8**
		**Oxygenated monoterpenes**	**79.2**	**88.7**
		**Sesquiterpene hydrocarbons**	-	-
		**Oxygenated sesquiterpenes**	-	**0.1**
		**Others**	**0.4**	**0.3**
		**Total identified**	**99.2**	**99.9**

^a^ Experimentally determined relative retention indices using a homologous series of n-alkanes (C_8_–C_20_) on the HP-INNOWAX column. ^b^ Trace (<0.1%) identification methods: MS; by comparison of the mass spectrum with those of the computer mass libraries Wiley, Adams, and NIST 08; RI: by comparison of the retention index with literature data [37,38,39].

**Table 2:** The results of antifungal activity *O. majorana* EOs.

Strains	OMN		OMC		Amphotericin B
	IC_50_	IC_90_	IC_50_	IC_90_	IC_90_
*C. albicans* ATCC 90028	0.0625	0.125	0.0625	0.125	0.01
*C. albicans* MFBF 10778 *	0.0625	0.50	0.0625	0.50	0.01
*C. albicans* MFBF 11100 **	0.0625	0.25	0.0625	0.25	0.01
*C. tropicalis* ATCC 750	0.125	0.50	0.25	0.50	0.25
*C. krusei* ATCC 14243	0.125	0.50	0.25	0.50	0.25
*C. dubliniensis* MFBF 11098	<0.0156	<0.0156	<0.0156	<0.0156	0.01

OMN: essential oil obtained from naturally grown *O. majorana*; OMC: essential oil obtained from cultivated *O. majorana*; * *Fluconazole-sensitive strain*; ** *Fluconazole-resistant strain.* Data presented as the mean of two measurements in µg mL^−1^.

**Table 3:** Germ-tube inhibition of *C. albicans* ATCC 90,023 treated with *O. majorana* EOs.

Media	OMN		OMC	
	0.0625 µg mL^−1^	0.125 µg mL^−1^	0.0625 µg mL^−1^	0.125 µg mL^−1^
YPG + 10% FBS	27 ± 2.94	38 ± 5.04	54 ± 4.36	51 ± 1.49
N-Acetyl-D-Glucosamine	48 ± 1.95	60 ± 2.78	58 ± 1.35	46 ± 2.28
Spider	81 ± 2.70 *	75 ± 3.28 *	83 ± 3.75 *	83 ± 5.62 *

OMN: essential oil from naturally harvested *O. majorana*; OMC: essential oil obtained from cultivated *O. majorana*; YPD: yeast potato glucose broth; FBS: fetal bovine serum. Data presented as a percentage as the mean ± SD on 100 *C. albicans* cells; * *p* < 0.05.

**Table 4:** Modulation of CSH by *O*. *majorana* EOs.

Sample	Concentration (µg mL^−1^)	Hydrophobicity Index	Inhibition of CSH (%)
OMN	0.125	11.19	52.61 *
	0.0625	10.44	58.41 *
OMC	0.125	18.65	25.70
	0.0625	23.13	7.85
Negative control	-	25.10	-

OMN: essential oil from naturally harvested *O. majorana*; OMC: essential oil obtained from cultivated *O. majorana*; * *p* < 0.05 in comparison to OMC.

## Data Availability

The data presented in this study are available in the article.

## Sample Availability

Samples of the EOs are available from the authors.

## References

[1] Swamy M.K., Akhtar M.S., Sinniah U.R. (2016). Antimicrobial properties of plant essential oils against human pathogens and their mode of action: An updated review. Evid. Based Complement. Altern. Med..

[2] Kaiser A., Carle R., Kammerer D.R. (2013). Effects of blanching on polyphenol stability of innovative paste-like parsley (*Petroselinum crispum* (Mill.) Nym ex A. W. Hill) and marjoram (*Origanum majorana* L.) products. Food Chem..

[3] FDA, Food and Drug Administration Electronic Code of Federal Regulations (eCFR). https://www.accessdata.fda.gov/scripts/cdrh/cfdocs/cfcfr/CFRSearch.cfm?fr=182.20.

[4] Febriani Y., Levallois P., Gingras S., Gosselin P., Majowicz S.E., Fleury M.D. (2010). The association between farming activities, precipitation, and the risk of acute gastrointestinal illness in rural municipalities of Quebec, Canada: A cross-sectional study. BMC Public Health.

[5] El Asbahani A., Miladi K., Badri W., Sala M., Aït Addi E.H., Casabianca H., El Mousadik A., Hartmann D., Jilale A., Renaud F.N.R. (2015). Essential oils: From extraction to encapsulation. Int. J. Pharm..

[6] Khan S.T., Khan M., Ahmad J., Wahab R., Abd-Elkader O.H., Musarrat J., Alkhathlan H.Z., Al-Kedhairy A.A. (2017). Thymol and carvacrol induce autolysis, stress, growth inhibition and reduce the biofilm formation by Streptococcus mutans. AMB Express.

[7] Feyaerts A.F., Mathé L., Luyten W., De Graeve S., Van Dyck K., Broekx L., Van Dijck P. (2018). Essential oils and their components are a class of antifungals with potent vapour-phase-mediated anti-Candida activity. Sci. Rep..

[8] Roby M., Sarhan M.A., Selim K.A.-H., Khalel K.I. (2013). Evaluation of antioxidant activity, total phenols and phenolic compounds in thyme (*Thymus vulgaris* L.), sage (*Salvia officinalis* L.), and marjoram (*Origanum majorana* L.) extracts. Ind. Crops Prod..

[9] Kirimer N., Başer K.H.C., Tümen G. (1995). Carvacrol-rich plants in Turkey. Chem. Nat. Compd..

[10] Rodriguez-Garcia I., Silva-Espinoza B., Ortega-Ramirez L., Leyva J., Siddiqui M.W., Valenzuela M.R.C., Gonzalez-Aguilar G., Zavala J.F.A. (2015). Oregano Essential Oil as an Antimicrobial and Antioxidant Additive in Food Products. Crit. Rev. Food Sci. Nutr..

[11] Tabanca N., Ozek T., Baser K.H.C., Tumen G. (2004). Comparison of the essential oils of *Origanum majorana* L. and *Origanum* × *majoricum* Cambess. J. Essent. Oil Res..

[12] Baser K.H.C., Özek T., Tümen G., Sezik E. (1993). Composition of the essential oils of Turkish Origanum species with commercial importance. J. Essent. Oil Res..

[13] Baser K.H.C., Kurkcuoglu M., Demirci B., Ozek T. (2003). The essential oil of *Origanum syriacum* L. var. sinaicum (Boiss.) Letswaart. Flav. Fragr. J..

[14] Erdogan A., Ozkan A. (2017). Investigation of Antioxidative, Cytotoxic, Membrane-Damaging and Membrane-Protective Effects of The Essential Oil of *Origanum majorana* and its Oxygenated Monoterpene Component Linalool in Human-Derived Hep G2 Cell Line. Iran. J. Pharm. Res..

[15] do Socorro Barbosa Chaves R., Martins R., Rodrigues A.B.L., de Menezes Rabelo E., Farias A.L.F., da Conceição Vieira Araújo C.M., Sobral T.F., Galardo A.K.R. (2019). Larvicidal Evaluation of the *Origanum majorana* L. Essential Oil against the Larvae of the Aedes aegypti Mosquito. bioRxiv.

[16] Ragab T.I., El Gendy A.N.G., Saleh I.A., Esawy M.A. (2019). Chemical Composition and Evaluation of Antimicrobial Activity of the *Origanum majorana* Essential Oil Extracted by Microwave-assisted Extraction, Conventional Hydro-distillation and Steam distillation. J. Essent. Oil Bear. Plants.

[17] Charai M., Mosaddak M., Faid M. (1996). Chemical Composition and Antimicrobial Activities of Two Aromatic Plants: Ori-ganum majorana L. and O. compactum Benth. J. Essent. Oil Res..

[18] Bouyahya A., Chamkhi I., Benali T., Guaouguaou F.-E., Balahbib A., El Omari N., Taha D., Belmehdi O., Ghokhan Z., El Menyiy N. (2020). Traditional use, phytochemistry, toxicology, and pharmacology of *Origanum majorana* L. J. Ethnopharmacol..

[19] Khadhri A., Bouali I., Aouadhi C., Lagel M.-C., Masson E., Pizzi A. (2019). Determination of phenolic compounds by MALDI–TOF and essential oil composition by GC–MS during three development stages of *Origanum majorana* L. Biomed. Chromatogr..

[20] Aladağ M.O., Özcan M.M., Ergin S. (2021). Inhibitory effect of some spice essential oils on growth of some gram-negative and gram-positive bacteria and a yeast. J. Food Process. Preserv..

[21] Omara S.T., El-Moez S.I., Mohamed A.M. (2014). Antibacterial Effect of *Origanum majorana* L. (Marjoram) and *Rosmarinus officinalis* L. (Rosemary) Essential Oils on Food Borne Pathogens Isolated from Raw Minced Meat in Egypt. Glob. Vet..

[22] Athamneh K., Alneyadi A., Alsamri H., Alrashedi A., Palakott A., El-Tarabily K.A., Eid A.H., Al Dhaheri Y., Iratni R. (2020). *Origanum majorana* Essential Oil Triggers p38 MAPK-Mediated Protective Autophagy, Apoptosis, and Caspase-Dependent Cleavage of P70S6K in Colorectal Cancer Cells. Biomolecules.

[23] Pimple P., Kadam P.V., Patil M.J. (2012). Ulcer healing properties of different extracts of *Origanum majorana* in streptozoto-cin-nicotinamide induced diabetic rats. Asian Pac. J. Trop.Disease.

[24] Cutuli M.A., Petronio G.P., Vergalito F., Magnifico I., Pietrangelo L., Venditti N., Di Marco R. (2019). *Galleria mellonella* as a consolidated in vivo model hosts: New developments in antibacterial strategies and novel drug testing. Virulence.

[25] Cotter G., Doyle S., Kavanagh K. (2000). Development of an insect model for the in vivo pathogenicity testing of yeasts. FEMS Immunol. Med. Microbiol..

[26] Fallon J., Kelly J., Kavanagh K. (2012). *Galleria mellonella* as a Model for Fungal Pathogenicity Testing. Host Fungus Interact..

[27] Sardi J.D.C.O., Polaquini C.R., Freires I.A., Galvao L.C.D.C., Lazarini J.G., Torrezan G.S., Regasini L.O., Rosalen P.L. (2017). Antibacterial activity of diacetylcurcumin against Staphylococcus aureus results in decreased biofilm and cellular adhesion. J. Med. Microbiol..

[28] Desbois A., Coote P.J. (2011). Wax moth larva (*Galleria mellonella*): An in vivo model for assessing the efficacy of antistaphylococcal agents. J. Antimicrob. Chemother..

[29] Harding C.R., Schroeder G., Collins J.W., Frankel G. (2013). Use of *Galleria mellonella* as a Model Organism to Study Legionella pneumophila Infection. J. Vis. Exp..

[30] Tuncsoy B.S., Tuncsoy M., Gomes T., Sousa V.S., Teixeira M.R., Bebianno M.J., Ozalp P. (2019). Effects of Copper Oxide Nanoparticles on Tissue Accumulation and Antioxidant Enzymes of *Galleria mellonella* L. Bull. Environ. Contam. Toxicol..

[31] Lionakis M.S. (2011). Drosophila and Galleria insect model hosts New tools for the study of fungal virulence, pharmacology and immunology. Virulence.

[32] Kavanagh K., Reeves E.P. (2004). Exploiting the potential of insects for in vivo pathogenicity testing of microbial pathogens. FEMS Microbiol. Rev..

[33] Cools F., Torfs E., Aizawa J., Vanhoutte B., Maes L., Caljon G., Delputte P., Cappoen D., Cos P. (2019). Optimization and Characterization of a *Galleria mellonella* Larval Infection Model for Virulence Studies and the Evaluation of Therapeutics Against Streptococcus pneumoniae. Front. Microbiol..

[34] Karaman M., Alvandian A., Bahar H. (2017). *Galleria mellonella* Larva Model in Evaluating the Effects of Biofilm in *Candida albicans*. Mikrobiyoloji Bulteni.

[35] Loh J.M.S., Adenwalla N., Wiles S., Proft T. (2013). *Galleria mellonella* larvae as an infection model for group A streptococcus. Virulence.

[36] Tsai C.J.-Y., Loh J.M.S., Proft T. (2016). *Galleria mellonella* infection models for the study of bacterial diseases and for antimicrobial drug testing. Virulence.

[37] Babushok V.I., Linstrom P.J., Zenkevich I.G. (2011). Retention Indices for Frequently Reported Compounds of Plant Essential Oils. J. Phys. Chem. Ref. Data.

[38] Yiğit Hanoğlu D., Hanoğlu A., Güvenir M., Süer K., Demirci B., Başer K.H.C., Özkum Yavuz D. (2017). Chemical composition and antimicrobial activity of the essential oil of Sideritis cypria Post endemic in Northern Cyprus. J. Essent. Oil Res..

[39] Kaskatepe B., Yıldız S.S., Kiymaci M.E., Yazgan A.N., Cesur S., Erdem S.A., Yıldız S.S. (2017). Chemical composition and antimicrobial activity of the commercial Origanum onites L. oil against nosocomial carbapenem resistant extended spectrum beta lactamase producer Escherichia coli isolates. Acta Biol. Hung..

[40] Amor G., Caputo L., La Storia A., De Feo V., Mauriello G., Fechtali T. (2019). Chemical Composition and Antimicrobial Activity of Artemisia herba-alba and *Origanum majorana* Essential Oils from Morocco. Molecules.

[41] Aytaç E. (2020). Comparison Essential Oil Contents *Origanum majorana* L. Obtained by Clevenger and SFE. HJBC..

[42] Busatta C., Vidal R., Popiolski A., Mossi A., Dariva C., Rodrigues M., Corazza F., Corazza M.L., Oliveira J.V., Cansian R. (2008). Application of *Origanum majorana* L. essential oil as an antimicrobial agent in sausage. Food Microbiol..

[43] Mossa A.-T., Nawwar G. (2011). Free radical scavenging and antiacetylcholinesterase activities of *Origanum majorana* L. essential oil. Hum. Exp. Toxicol..

[44] Baser K., Kirimer N., Tümen G. (1993). Composition of the Essential Oil of *Origanum majorana* L. from Turkey. J. Essent. Oil Res..

[45] Tajkarimi M.M., Ibrahim S.A., Cliver D.O. (2010). Antimicrobial herb and spice compounds in food. Food Control.

[46] Pina-Vaz C., Gonçalves Rodrigues A., Pinto E., Costa-de-Oliveira S., Tavares C., Salgueiro L., Cavaleiro C., Gonçalves M.J., Martinez-de-Oliveira C. (2004). Antifungal activity of *Thymus* oils and their major compounds. J. Eur. Acad. Derma Vener.

[47] Lima I.O., Pereira F.D.O., Oliveira W.A.D., Lima E.D.O., Menezes E.A., Cunha F.A., Diniz M.D.F.F.M. (2013). Antifungal activity and mode of action of carvacrol against *Candida albicans* strains. J. Essent. Oil Res..

[48] Raut J.S., Shinde R., Chauhan N., Karuppayil S.M. (2012). Terpenoids of plant origin inhibit morphogenesis, adhesion, and biofilm formation by *Candida albicans*. Biofouling.

[49] Suntres Z.E., Coccimiglio J., Alipour M. (2015). The Bioactivity and Toxicological Actions of Carvacrol. Crit. Rev. Food Sci. Nutr..

[50] Hajlaoui H., Mighri H., Aouni M., Gharsallah N., Kadri A. (2016). Chemical composition and in vitro evaluation of antioxidant, antimicrobial, cytotoxicity and anti-acetylcholinesterase properties of Tunisian *Origanum majorana* L. essential oil. Microb. Pathog..

[51] Sarer E., Scheffer J.J.C., Janssen A.M., Svendsen A.B., Svendsen A.B., Scheffer J.J.C. (1985). Composition of the essential oil of *Origanum majorana* grown in different localities in Turkey. Essential Oils and Aromatic Plants.

[52] Hacioglu M., Oyardi O., Kirinti A. (2021). Oregano essential oil inhibits *Candida* spp. biofilms. Z. Nat. C.

[53] Calderone R.A., Fonzi W.A. (2001). Virulence factors of *Candida albicans*. Trends Microbiol..

[54] Mayer F.L., Wilson D., Hube B. (2013). *Candida albicans* pathogenicity mechanisms. Virulence.

[55] El-Baz A., Mosbah R., Goda R., Mansour B., Sultana T., Dahms T., El-Ganiny A. (2021). Back to Nature: Combating *Candida albicans* Biofilm, Phospholipase and Hemolysin Using Plant Essential Oils. Antibiotics.

[56] McCullough M., Ross B., Reade P. (1996). *Candida albicans*: A review of its history, taxonomy, epidemiology, virulence attributes, and methods of strain differentiation. Int. J. Oral Maxillofac. Surg..

[57] Haynes K. (2001). Virulence of Candida species. Trends Microbiol..

[58] Mroczyńska M., Brillowska-Dąbrowska A. (2021). Virulence of Clinical *Candida* Isolates. Pathogens.

[59] Midkiff J., Borochoff-Porte N., White D., Johnson D.I. (2011). Small Molecule Inhibitors of the *Candida albicans* Budded-to-Hyphal Transition Act through Multiple Signaling Pathways. PLoS ONE.

[60] Palmeira-De-Oliveira A., Salgueiro L., Palmeira-De-Oliveira R., De Oliveira J.M., Pina-Vaz C., Queiroz J., Rodrigues A.G. (2009). Anti-Candida Activity of Essential Oils. Mini Rev. Med. Chem..

[61] Bujdáková H., Didiášová M., Drahovská H., Černáková L. (2013). Role of cell surface hydrophobicity in *Candida albicans* biofilm. Open Life Sci..

[62] Vertyporokh L., Wojda I. (2020). Immune response of *Galleria mellonella* after injection with non-lethal and lethal dosages of *Candida albicans*. J. Invertebr. Pathol..

[63] Katragkou A., Kruhlak M.J., Simitsopoulou M., Chatzimoschou A., Taparkou A., Cotten C.J., Paliogianni F., Diza-Mataftsi E., Tsantali C., Walsh T.J. (2010). Interactions between Human Phagocytes and *Candida albicans* Biofilms Alone and in Combination with Antifungal Agents. J. Infect. Dis..

[64] Fuchs B.B., Eby J., Nobile C., El Khoury J.B., Mitchell A.P., Mylonakis E. (2010). Role of filamentation in *Galleria mellonella* killing by *Candida albicans*. Microbes Infect..

[65] CLSI (2012). Performance Standards for Antimicrobial Susceptibility Testing.

[66] Zoric N., Horvat I., Kopjar N., Vucemilovic A., Kremer D., Tomic S., Kosalec I. (2013). Hydroxytyrosol Expresses Antifungal Activity In Vitro. Curr. Drug Targets.

[67] Zuzarte M., Goncalves M.J., Cavaleiro C., Canhoto J., Vale-Silva L., Silva M.J., Pinto E., Salgueiro L. (2011). Chemical composition and antifungal activity of the essential oils of Lavandula viridis L’Her. J. Med. Microbiol..

[68] Ishida K., Palazzo de Mello J.C., Cortez D.A.G., Filho B.P.D., Ueda-Nakamura T., Nakamura C.V. (2006). Influence of tannins from Stryphnodendron adstringens on growth and virulence factors of *Candida albicans*. J. Antimi Crob. Chemother..

[69] Wijesinghe G.K., Maia F.C., De Oliveira T.R., De Feiria S.N.B., Joia F., Barbosa J.P., Boni G.C., Sardi J.D.C.O., Rosalen P.L., Höfling J.F. (2020). Effect of Cinnamomum verum Leaf Essential Oil on Virulence Factors of Candida Species and Determination of the In-Vivo Toxicity with *Galleria mellonella* Model. Mem. Inst. Oswaldo Cruz.

[70] Çim S., Altuntaş H. (2021). Anti-oxidative, genotoxic and mutagenic effects of idiobiont, endoparasitoid, Pimpla turionellae L. (Hymenoptera: Ichneumonidae) venom on its host *Galleria mellonella* L. (Lepidoptera: Pyralidae). Biol. Control.

[71] Wu G., Liu Y., Ding Y., Yi Y. (2016). Ultrastructural and functional characterization of circulating hemocytes from *Galleria mellonella* larva: Cell types and their role in the innate immunity. Tissue Cell.

